# Impact of fixed orthodontic appliances on day-to-day life experiences among adolescents: a cross-sectional observational study.

**DOI:** 10.1186/s12903-026-09072-1

**Published:** 2026-07-06

**Authors:** Shimaa Mohamed G. Ahmed, Nada O. El-Zawahry, Fouad Aly El-Sharaby

**Affiliations:** Department of Orthodontic, Faculty of Dentistry, Cairo University, Giza, 2017 Egypt

**Keywords:** Malocclusion, Fixed orthodontic appliances, Oral health-related quality of life, Pain and discomfort, Adolscents, Social and emotional well-being

## Abstract

**Background:**

Fixed orthodontic appliances are widely used for the correction of malocclusions in adolescents. Although effective in achieving optimal dental alignment and occlusion, they may influence patients’ daily life experiences. This study aimed to assess the impact of fixed orthodontic appliances on oral function, pain, discomfort, and social and emotional well-being among adolescents.

**Materials and methods:**

A cross-sectional observational study was conducted on 364 Egyptian adolescents aged 13–18 years (242 females, 122 males). Participants were categorized according to the type of orthodontic appliance: fixed labial appliances only (*n* = 235), labial appliances with mini-screws (*n* = 67), labial appliances with palatal/lingual components (*n* = 44), combined mini-screws and palatal/lingual appliances (*n* = 12), and fixed retainers (*n* = 6). All participants completed the Arabic version of the Orthodontic Treatment Impact Questionnaire (OTIQ), which evaluates oral function, pain, discomfort, and social-emotional well-being. Data were analyzed using Mann–Whitney U, Kruskal–Wallis, and Spearman correlation tests.

**Results:**

Most participants reported minimal impairment in oral function. Social and emotional well-being was largely unaffected, with 90.1% expressing no concern about appearance and 87.4% reporting no experience of teasing. Pain and discomfort were generally moderate, with 40.7% reporting jaw ache and 60.7% experiencing food impaction. Appliance type significantly influenced outcomes: combined labial appliances with mini-screws and palatal/lingual components were associated with the highest levels of discomfort, whereas fixed retainers showed the lowest impact. Labial appliances were more frequently associated with social concerns. No significant gender differences were observed. A significant negative correlation was found between age and social well-being (*p* < 0.001), while treatment duration was positively correlated with pain and discomfort (*p* = 0.018).

**Conclusion:**

Fixed orthodontic appliances had minimal impact on oral function and social-emotional well-being in adolescents. Patients with labial appliances combined with mini-screws and palatal/lingual appliances reported more pain than those with retainers. Longer treatment duration was linked to greater pain.

**Supplementary Information:**

The online version contains supplementary material available at 10.1186/s12903-026-09072-1.

## Background

Malocclusion is a highly prevalent oral health condition that often requires orthodontic intervention for its correction [1]. Its prevalence is reported to be highest during the early stages of dentition, reaching approximately 54% in deciduous dentition and remaining at a similar level in permanent dentition. These findings highlight malocclusion as a significant public health concern, not only due to its clinical implications but also because of its associated economic burden on families and healthcare systems [2].

Orthodontic treatment is primarily aimed at improving dental esthetics and function, which can subsequently enhance an individual’s social appearance, self-esteem, and overall well-being. In this context, oral health-related quality of life (OHRQoL) has emerged as an important outcome measure, reflecting the impact of oral and dental conditions on an individual’s daily functioning and psychosocial status [3].

Fixed orthodontic appliances remain the most used modality for the correction of malocclusions, particularly among adolescents. While these appliances are effective in achieving optimal alignment and occlusion, they may also influence patients’ day-to-day experiences. Commonly reported effects include pain and discomfort, especially following appliance placement and periodic adjustments. Such symptoms may interfere with routine activities, including eating, and often necessitate temporary dietary modifications [4].

Despite their therapeutic benefits, orthodontic appliances can impose physical and psychosocial challenges during treatment. Therefore, adequate support from orthodontic professionals, as well as from family and peers, is essential to help adolescents adapt to these changes and maintain their oral health and overall well-being throughout the course of treatment [5].

To date, there is a lack of published data evaluating the impact of orthodontic treatment on the daily life experiences of Egyptian adolescents undergoing treatment with different types of fixed orthodontic appliances. Addressing this gap is important for understanding patient-centered outcomes within this population. Therefore, the present cross-sectional observational questionnaire-based study aimed to assess the impact of fixed orthodontic appliances on adolescents’ social and emotional well-being, as well as to evaluate their effects on oral function, pain, and discomfort.

## Materials and methods

This investigation was designed as an observational, descriptive, cross-sectional study. It was conducted at the Department of Orthodontics, Faculty of Dentistry, Cairo University, Egypt, over a period of five months, from August to December 2023.

The target population consisted of adolescent orthodontic patients between 13 and 18 years of age. Patients were recruited consecutively as they attended their routine follow-up visits at the orthodontic clinic.

Sample size was calculated prior to recruitment using StatCalc software from Epi Info™ (Centers for Disease Control and Prevention, USA). The calculation was based on the assumption of a 62% expected improvement rate in treatment-related quality of life reported in a previous study by Benson et al. [5] Setting the confidence level at 95% and the margin of error at 5%, the minimum required sample size was determined to be 362. To account for possible non-response or incomplete questionnaires, 380 patients were initially targeted, and the final number of completed responses was 364, which exceeded the calculated requirement.

The research Ethics Committee of Faculty of Dentistry - Cairo University provided ethical approval before starting the study (Approval No.: 27723). All participants, and where appropriate their parents or guardians, were provided with detailed information about the study objectives, procedures, potential benefits, and possible risks. Written informed consent was obtained from each participant prior to inclusion. Patients were assured that their participation was voluntary, that they could withdraw at any stage without consequence to their treatment, and that confidentiality of their responses would be strictly maintained.

Regarding patients’ criteria; Adolescents aged 13 to 18 years, Male or female patients undergoing orthodontic treatment with fixed labial appliances (with or without mini-screws or palatal/lingual appliances) or wearing fixed retainers. Patients willing to participate and able to complete the questionnaire were included. While Patients with cleft lip and/or palate or other craniofacial anomalies, Illiterate patients or those unable to comprehend the questionnaire, Patients with complicated medical histories that could confound results, those undergoing combined orthodontic–surgical treatment and Patients or guardians refusing consent were excluded.

Among participants who completed the questionnaire: patients with fixed labial appliance only (*n* = 235), patients with fixed labial appliance combined with mini-screws (*n* = 67), patients with fixed labial appliance combined with palatal/lingual appliance (*n* = 44), patients with fixed labial appliance combined with both mini-screws and palatal/lingual appliance (*n* = 12) and patients with fixed retainer (*n* = 6).

Clinical examination was conducted to confirm the type of orthodontic appliance being used. Following classification into the appropriate appliance group, each participant was asked to complete the validated Arabic version of the OTIQ in person just before the scheduled orthodontic activation appointment to standardize the timing of assessment among participants. A trained examiner (SM) was present to provide clarification if required, while ensuring that responses remained self-reported to minimize interviewer bias.

The OTIQ is a validated patient-reported outcome measure originally developed by Benson et al. [5]. It consists of 22 items grouped into three major domains: Impact on daily life and social–emotional well-being (e.g., embarrassment, teasing, concerns about appearance). Sensation in the mouth (e.g., food trapping, difficulty cleaning, speech interference) in addition to feelings and perceptions related to appliances (e.g., pain, jaw ache, discomfort). Each item is scored on a three-point Likert scale (0 = no impact, 1 = moderate impact, 2 = severe impact). Items were scored on a three-point Likert scale (0–2). Higher scores indicated greater negative impact for negatively worded items. Positively worded items, including ‘I feel normal’ and ‘I feel attractive,’ were not reverse-scored and indicated less negative impact. Accordingly, interpretation of the social and emotional well-being domain should be made with caution. Since the original OTIQ was in English, the questionnaire was translated, culturally adapted into Arabic. The validation was conducted as a part of the present study. The original English version of the OTIQ is provided as a Supplementary File and the final validated Arabic version used in this study is provided as Supplementary File 2. The translation process followed international guidelines for cross-cultural adaptation of self-reported measures [6]. The steps included: Forward translation by two bilingual translators (one dental professional and one non-medical translator). Synthesis of the translations into a single Arabic draft. Backward translation into English by two independent translators blinded to the original questionnaire. Expert panel review consisting of orthodontists, methodologists, and language experts to resolve discrepancies and ensure semantic, idiomatic, experiential, and conceptual equivalence. Pretesting and cognitive interviewing with 36 patients to confirm clarity and cultural appropriateness. Reliability testing showed good internal consistency with Cronbach’s α > 0.8 for all domains. Test–retest reliability, assessed after two weeks in 30 patients, demonstrated acceptable intraclass correlation coefficients (> 0.75), confirming stability of the instrument.

Several strategies were employed to minimize bias: Selection bias: All consecutive eligible patients were invited to participate, ensuring representative sampling. Information bias: The purpose of the study was explained without emphasizing specific outcomes to avoid influencing responses. Reporting bias: All outcomes assessed by the questionnaire were reported, avoiding selective reporting. Interviewer bias: Questionnaires were self-administered, with minimal investigator interference.

### Statistical analysis

Data was entered and analyzed using SPSS software version 23.0 (IBM Corp., Armonk, NY, USA). The normality of continuous data (age, treatment duration, and OTIQ scores) was evaluated using the Shapiro-Wilk test. Categorical variables (e.g., gender, type of appliance) were presented as frequencies and percentages. Because the continuous variables deviated significantly from a normal distribution, they were summarized using medians and ranges; however, mean and standard deviation (SD) values were also provided for descriptive purposes. Accordingly, non-parametric analyses were employed: the Kruskal-Wallis test was used to compare questionnaire scores across different orthodontic appliances, followed by adjusted pairwise comparisons where significant, while the Mann-Whitney U test was used to evaluate differences between genders. Statistical significance was set at *p* ≤ 0.05. Internal consistency and reliability of the questionnaire responses within the current sample were reassessed and confirmed using Cronbach’s α.

## Results

After 380 adolescents were evaluated for eligibility, 16 declined to take part, leaving 364 participants in the final sample (122 males, 33.5%; 242 females, 66.5%). As shown in Table 1, the majority of subjects reported only little oral function impairment. Approximately two-thirds were not bothered by difficulty chewing or swallowing (67.3%), difficulty pronouncing words (68.7%), or cleaning braces/retainers (61.5%). The most frequently reported functional problem was difficulty eating certain foods, with 43.1% reporting moderate difficulty and 18.1% reporting severe difficulty. Most participants (82.7%) also reported no difficulty sleeping. The oral function score had a median of 2 (range: 0–8) and a mean ± SD of 2.3 ± 1.9. Social and emotional well-being was generally unaffected. The majority of participants were not concerned about the appearance of their appliance (90.1%) and did not report teasing (87.4%). In addition, 67.9% reported feeling normal during treatment, while only 1.9% reported feeling ugly. The social well-being score showed a median of 4 (range: 0–15) and a mean ± SD of 4.8 ± 2.6. Pain and discomfort scores were slightly higher than the other domains. Food impaction was the most frequently reported complaint, affecting 60.7% of participants moderately and 10.4% severely. Jaw ache was reported moderately by 40.7% and severely by 17% of participants. The pain and discomfort score had a median of 3 (range: 0–10) and a mean ± SD of 3.2 ± 2.2.

**Table 1:** Frequencies (n), percentages (%) and descriptive statistics for oral function, social well-being questions and pain and discomfort questions

Oral function	Doesn’t bother me		Bothers me a bit		Bothers me a lot	
	n	%	n	%	n	%
1. Difficulty chewing or swallowing.	245	67.3	82	22.5	37	10.2
2. Difficulty eating certain foods.	141	38.7	157	43.1	66	18.1
3. Difficulty pronouncing words.	250	68.7	92	25.3	22	6
4. Difficulty sleeping.	301	82.7	41	11.3	22	6
5. Difficulty cleaning my brace or retainer.	224	61.5	109	29.9	31	8.5
Scores						
Median (Range)	2 (0 – 8)					
Mean (SD)	2.3 (1.9)					

Social well-being	Doesn’t bother me		Bothers me a bit		Bothers me a lot	
	n	%	n	%	n	%
1. Worrying about my brace breaking.	187	51.4	105	28.8	72	19.8
2. The appearance of my brace or retainer.	328	90.1	24	6.6	12	3.3
3. Having my photograph taken.	281	77.2	55	15.1	28	7.7
4. Being teased.	318	87.4	39	10.7	7	1.9
	Don’t feel at all		Feel a bit		Feel a lot	
5. I feel annoyed.	269	73.9	81	22.3	14	3.8
6. I feel shy.	321	88.2	31	8.5	11	3
7. I feel normal.	37	10.2	80	22	247	67.9
8. I feel weird.	302	83	51	14	11	3
9. I feel ugly.	342	94	15	4.1	7	1.9
10. I feel attractive.	151	41.5	112	30.8	101	27.7
11. I feel negative about my smile.	269	73.9	72	19.8	23	6.3
Scores						
Median (Range)	4 (0 – 15)					
Mean (SD)	4.8 (2.6)					

Pain and discomfort	Doesn’t bother me		Bothers me a bit		Bothers me a lot	
	n	%	n	%	n	%
Food getting stuck in my brace or retainer.	105	28.8	221	60.7	38	10.4
Feeling tight.	231	63.5	108	29.7	25	6.9
Rubbing on my gums.	229	62.9	76	20.9	59	16.2
Making my jaw ache.	154	42.3	148	40.7	62	17
Catching the inside of my mouth.	180	49.5	115	31.6	69	19
Scores						
Median (Range)	3 (0 – 10)					
Mean (SD)	3.2 (2.2)					

Regarding the overall effect of orthodontic treatment on participants’ daily lives, 58.2% reported no effect, 36% reported a slight effect, and only 5.8% reported a major effect. The overall life impact score showed a median of 0 (range: 0–2) and a mean ± SD of 0.5 ± 0.6. The total questionnaire score had a median of 10 (range: 1–33) and a mean ± SD of 10.8 ± 5.7.

**Fig. 1:**
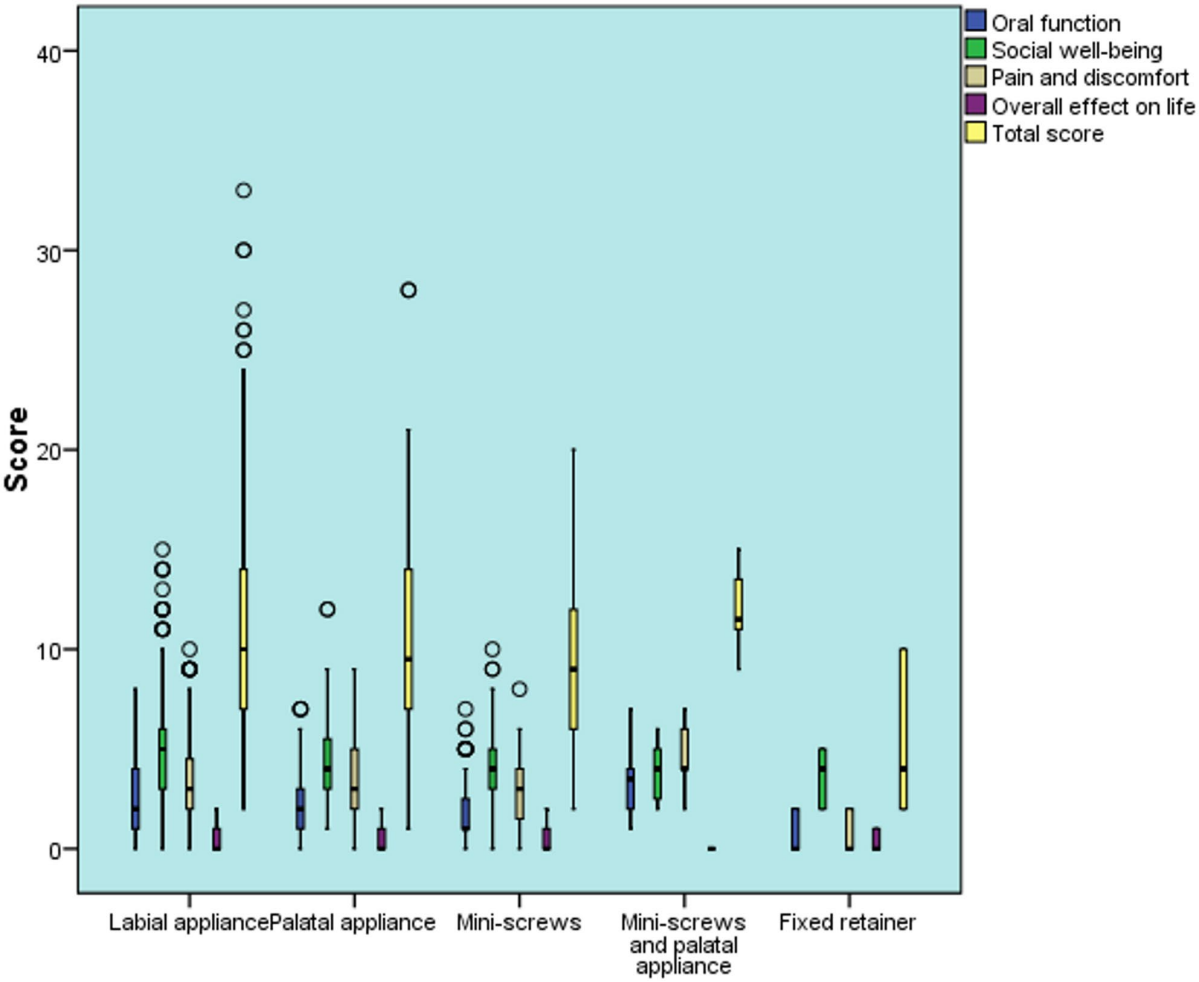
Box plot representing median and range values for questionnaire scores with different appliances (Circles represent outliers)

Significant differences with small effect sizes were found between appliance groups for oral function (*p* = 0.011), social well-being (*p* = 0.045), pain and discomfort (*p* = 0.003), overall life impact (*p* = 0.026), and total questionnaire score (*p* = 0.018). Participants treated with combined mini-screws and palatal/lingual appliances generally reported higher oral function and pain/discomfort scores, whereas fixed retainers showed low scores across most domains. Labial appliances demonstrated slightly higher social well-being scores than other appliance types. For total questionnaire scores (*p* = 0.018), labial appliances combined with mini-screws and palatal/lingual appliances scored the highest. Fixed retainer showed the statistically significant lowest score, as presented in Table 2 and in Fig. 1. However, findings related to the smaller appliance groups, particularly fixed retainers and combined appliance groups, should be interpreted cautiously because of the limited sample sizes.

**Table 2:** Descriptive statistics and results of Kruskal-Wallis test for the association between questionnaire scores with different appliances

Dimension	Labial appliance (n = 235)		palatal/lingual appliance (n = 44)		Mini-screws (n = 67)		Mini-screws and palatal/lingual appliance (n = 12)		Fixed retainer (n = 6)		P-value	Effect size (Eta squared)
	Median (Range)	Mean (SD)	Median (Range)	Mean (SD)	Median (Range)	Mean (SD)	Median (Range)	Mean (SD)	Median (Range)	Mean (SD)		
Oral function	2 (0-8) ^B^	2.3 (2)	2 (0-7) ^B^	2.6 (1.9)	1 (0-7) ^B^	2 (1.7)	3.5 (1-7) ^A^	3.5 (2)	0 (0-2) ^C^	0.7 (1)	0.011*	0.030
Social well-being	5 (0-15)^A^	5.1 (2.7)	4 (1-12)^B^	4.5 (2.4)	4 (0-10)^B^	4.1 (2.1)	4 (2-6) ^B^	3.9 (1.5)	4 (2-5)^B^	3.7 (1.4)	0.045*	0.018
Pain and discomfort	3 (0-10)^B^	3.3 (2.3)	3 (0-9)^B^	3.5 (2.1)	3 (0-8)^B^	3 (1.8)	4 (2-7) ^A^	4.5 (1.7)	0 (0-2)^C^	0.7 (1)	0.003*	0.038
Overall effect on life	0 (0-2)^A^	0.5 (0.6)	0 (0-2) ^A^	0.5 (0.7)	0 (0-2) ^A^	0.4 (0.6)	0 (0-0) ^B^	0 (0)	0 (0-1) ^A^	0.3 (0.5)	0.026*	0.026
Total score	10 (2-33)^B^	11.1 (6)	9.5 (1-28)^B^	11 (6.1)	9 (2-20)^B^	9.6 (4.1)	11.5 (9-15) ^A^	11.9 (1.8)	4 (2-10)^C^	5.3 (3.7)	0.018*	0.028

*: Significant at P ≤ 0.05, Different superscripts in the same row indicate statistically significant difference between appliances according to adjusted pairwise comparison test

**Fig. 2:**
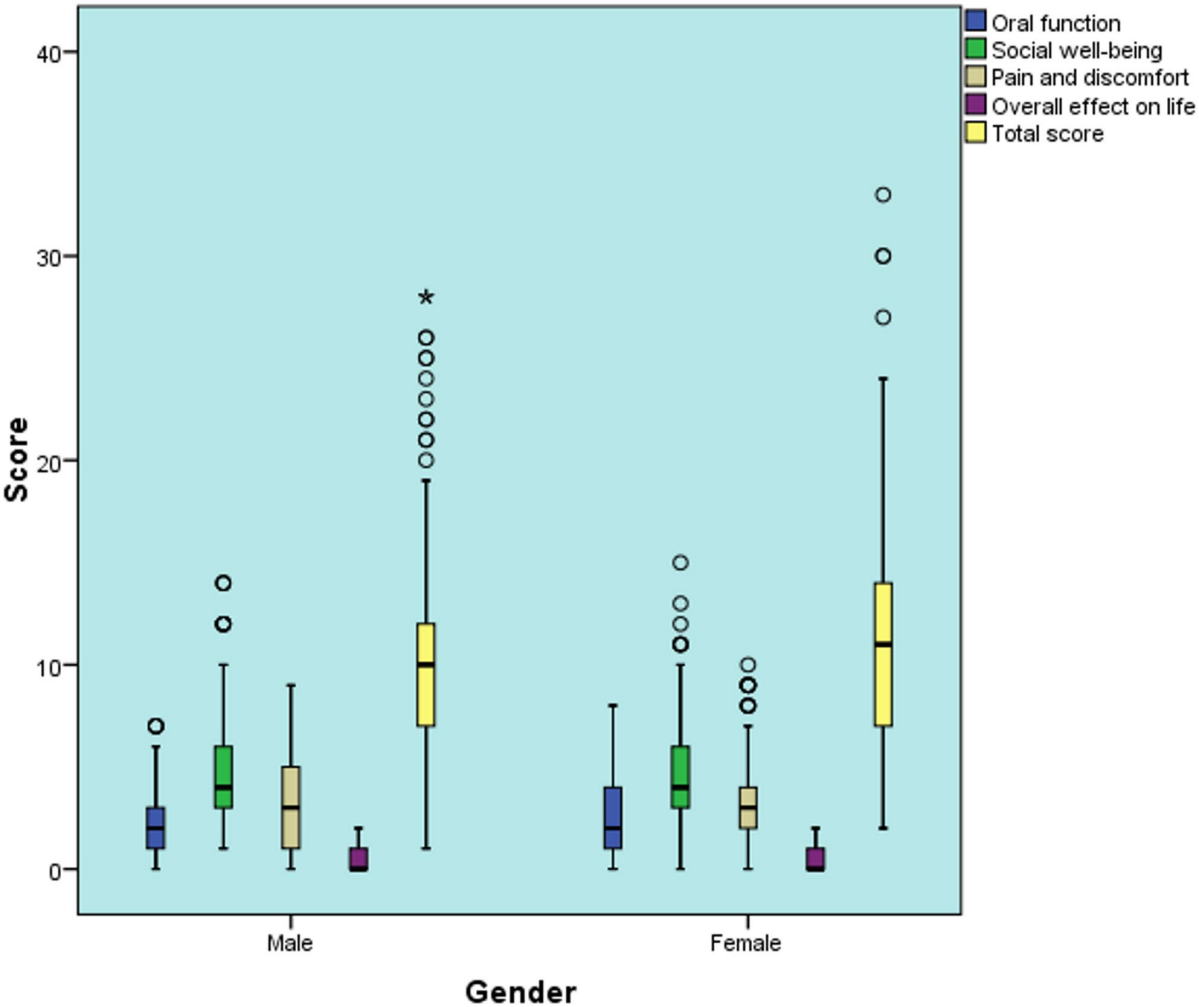
Box plot representing median and range values for questionnaire scores of males and females (Circles and stars represent outliers)

No statistically significant differences were found between males and females in oral function, social well-being, pain and discomfort, overall life impact, or total questionnaire scores (*p* > 0.05 for all comparisons) as shown in (Table 3) and (Fig. 2).

**Table 3:** Descriptive statistics and results of Mann-Whitney U test for the association between gender and questionnaire scores

Dimension	Males (n = 122)		Females (n = 242)		P-value	Effect size (d)
	Median (Range)	Mean (SD)	Median (Range)	Mean (SD)		
Oral function	2 (0-7)	2.1 (1.8)	2 (0-8)	2.4 (2)	0.253	0.118
Social well-being	4 (1-14)	4.7 (2.9)	4 (0-15)	4.8 (2.4)	0.178	0.14
Pain and discomfort	3 (0-9)	3 (2.3)	3 (0-10)	3.3 (2.2)	0.202	0.133
Overall effect on life	0 (0-2)	0.5 (0.6)	0 (0-2)	0.4 (0.6)	0.185	0.121
Total score	10 (1-28)	10.3 (6)	11 (2-33)	11 (5.5)	0.132	0.158

*: Significant at P ≤ 0.05

**Fig. 3:**
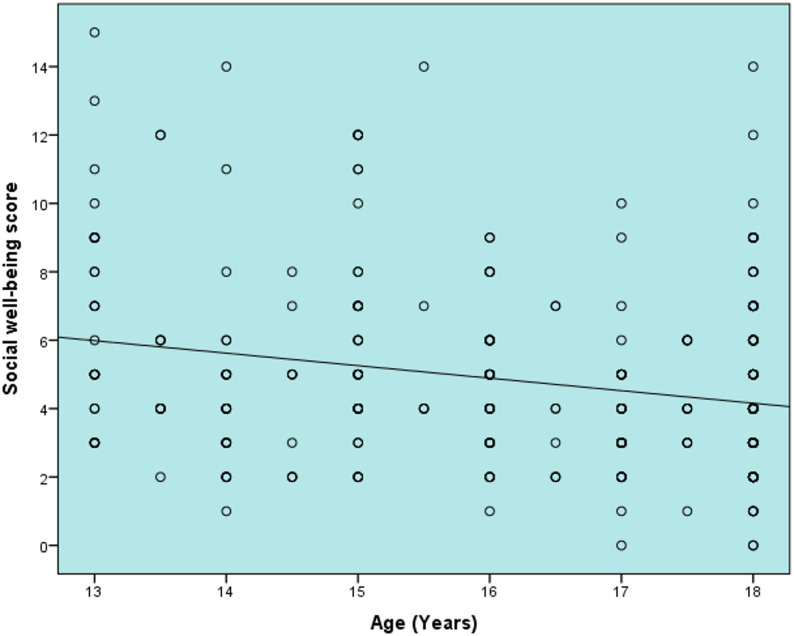
Scatter diagram representing inverse correlation between age and social and emotional well-being score

Age was not significantly correlated with oral function, pain/discomfort, overall life impact, or total questionnaire score (Fig. 3). However, a significant negative correlation was observed between age and social well-being scores (*r* = − 0.203, *p* < 0.001), indicating lower social well-being scores among older adolescents. However, cautious interpretation of this finding should be made because the domain included both positively and negatively worded items.

**Fig. 4:**
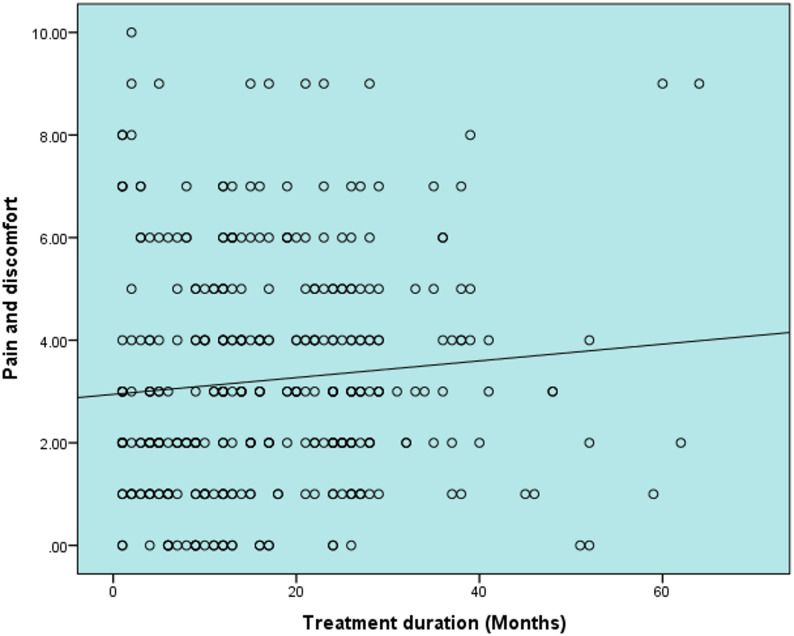
Scatter diagram representing direct correlation between treatment duration and pain and discomfort

The correlation between treatment duration and questionnaire score is presented in (Fig. 4). Treatment duration showed no significant correlation with oral function, social and emotional well-being, overall life impact, or total questionnaire scores. However, a significant positive correlation was found between treatment duration and pain/discomfort (*r* = 0.124, *p* = 0.018), indicating that longer treatment was associated with greater pain levels.

There was very good test re-test reliability with Cronbach’s alpha values ranging from 0.806 to 0.844.

## Discussion

Orthodontic treatment is primarily aimed at improving both dentofacial aesthetics and oral function, which in turn can enhance patients’ quality of life. Previous studies have shown that malocclusion may negatively affect individuals’ psychological and social well-being, and that its correction through orthodontic treatment can lead to improvements in oral health-related quality of life [7]. The present study explored how fixed orthodontic appliances influence the day-to-day life of adolescents, with particular attention to oral function, pain and discomfort, and social and emotional well-being.

The study population comprised adolescents aged 13–18 years, as this age group most commonly seeks orthodontic treatment. At this stage, most permanent teeth have erupted, and ongoing growth allows for effective orthodontic intervention [8]. Adolescence represents a transitional developmental stage characterized by ongoing cognitive and psychosocial maturation, making it a suitable period for assessing patient-reported outcomes using age-appropriate instruments. Researchers were able to create questionnaires tailored to this age group [5].

Including both male and female participants allowed for a more comprehensive and generalizable assessment of the impact of fixed orthodontic treatment on adolescents’ day-to-day life. This approach provided a broader understanding of patients’ perceptions regarding pain, discomfort, oral health, and psychosocial and emotional well-being. Previous research has suggested that females tend to report higher levels of pain and discomfort, while males may be less sensitive to aesthetic and social aspects of treatment. Such differences in perception may influence overall quality of life outcomes and patient satisfaction with orthodontic care [8].

Our study findings revealed that participants experienced little pain and discomfort during orthodontic treatment with a mean score of 3.2 (± 2.2) out of 10. This agreed with Banerjee et al. [9] who reported that 58% of participants complained of only mild pain.

Regarding difficulty in eating certain foods, 43% of participants reported being slightly affected, while 18% reported being significantly affected. This can be attributed to the mechanical interference from brackets and temporary changes in masticatory patterns after appliance placement. This finding is consistent with Carter et al. [10], who reported that adolescents undergoing treatment with fixed appliances often experience difficulties with chewing, take longer to complete meals, and may adopt dietary restrictions.

Although appliance type showed statistical differences in social and emotional well-being, yet limited clinical significance. Comparisons revealed that the fixed labial appliance only showed the statistically significant highest score. However, the overall impact of orthodontic treatment was minimal indicated little impact of fixed orthodontic appliances on the social and emotional well-being status of the participants. This suggests that adolescents may prioritize functional adaptation over aesthetic concerns during treatment, or that social acceptance of orthodontic appliances has improved in contemporary societies. In our study almost two-thirds of participants 67.9% felt normal which was in accordance with Nedzinskaitė et al. who reported that almost 50% of the subjects were feeling good and that about 38% were feeling normal [11].

In the present study, no significant differences were observed between males and females in terms of perceived pain and discomfort. This finding is consistent with previous research indicating that gender does not significantly influence pain perception during orthodontic treatment suggesting that biological responses to orthodontic forces may be relatively consistent across sexes [12]. However, another study reported higher pain levels among females [13], as well as findings by Jawaid et al. [14], who reported greater pain perception among males. The conflicting evidence in the literature indicated that psychosocial and cultural factors may influence pain reporting, highlighting the subjective and multifactorial nature of orthodontic discomfort.

Surprisingly, there was no statistically significant association between gender and social and emotional well-being. This finding was consistent with Toti et al. who assessed both males and females after 12 months of orthodontic treatment and there was no significant difference in the psychosocial impact [15]. However, Alqefari et al. [16]. stated that females had a higher impact on psychological aspects than males, this was mainly because females are more concerned about social appearance than males as many of them find the fixed metallic appliances unaesthetic.

The type of orthodontic appliance appeared to play a significant role in patients’ experiences. Combined appliances, particularly those involving mini-screws and palatal/lingual components, were associated with higher levels of discomfort and functional limitation. This may be explained by the increased bulk and complexity of these appliances, which can interfere more with oral functions or may cause soft tissue irritation. In contrast, fixed retainers were associated with lower levels of discomfort, likely due to patient adaptation and their relatively passive role following active treatment. Deteriorating OHRQoL was linked to debonding or fracture rather than the retainer presence [17, 18]. Although many comparisons reached statistical significance, the effect sizes were small, indicating limited differences between appliance groups. Therefore, the clinical significance of these findings should be interpreted cautiously. The rationale for including fixed retainers was that they still represent a fixed intraoral orthodontic component that may influence daily life experiences, oral comfort, speech, and oral hygiene. However, the authors acknowledge that patient experiences during retention differ from active treatment.

Anchorage is a fundamental component of orthodontic treatment and is required in most cases to control unwanted tooth movement [19]. Sharma et al. [20] reported that 32% of patients required a trans-palatal arch (TPA) and 26% required a lingual arch (LA), highlighting the frequent need for auxiliary anchorage devices. In the present study, among the 364 participants, 44 patients were treated with palatal/lingual appliances, 67 with mini-screws, and 12 with a combination of both.

Temporary anchorage devices (TADs) are widely accepted in contemporary orthodontics; however, their use remains selective depending on treatment requirements. In our sample, 79 adolescents (21.7%) received treatment involving TADs. This finding is consistent with Hsin-Chung Cheng et al. [21], who reported that 47.1% of orthodontists use TADs in more than half of their cases. In contrast, lower utilization rates have been reported in other regions, such as India, where approximately 43.7% of orthodontists incorporate TADs into their practice [22].

Regarding the effect of the braces or the retainers on patients’ overall lives, more than half of the participants reported that the braces or retainers didn’t affect their lives, and only 5.8% of the sample reported that their lives were affected a lot. This finding suggested that adolescents may rapidly adapt to orthodontic appliances in their daily lives. This adaptation process may decrease the long-term perceived burden although there was initial discomfort at the beginning of the treatment. This was in accordance with another study who concluded that 38% reported that their overall life was not affected at all and almost 8% of the participants reported that their brace affected their overall life a lot [5].

An important finding of this study was the significant negative correlation between age and social well-being domain scores. However, interpretation of this association should be approached cautiously, as the domain included both positively and negatively worded items that were not reverse-scored. This may be due to increased social awareness and sensitivity to appearance during later adolescence. Additionally, a positive correlation was observed between treatment duration and pain/discomfort, suggesting that prolonged treatment may contribute to cumulative discomfort. This finding was in line with Baseer et al., 2021 [23] who reported a significant positive correlation between the length of orthodontic treatment and pain intensity.

Finally, regarding the total questionnaire score, there was a statistically significant difference between all the appliances. Comparisons revealed that fixed labial appliances associated with mini-screws and palatal/lingual appliances showed the statistically significant high scores. In contrast, fixed retainers showed the statistically significant low scores. However, these differences should be interpreted cautiously, as they might not result in significant changes in the day-to-day functioning of patients due to the very small impact sizes.

Several limitations should be considered in this study. The cross-sectional design and reliance on self-reported responses limit causal interpretation and may be influenced by individual perceptions. Additionally, participants were recruited from a single clinic, which may affect generalizability. Moreover, potential confounding factors such as treatment complexity, severity of malocclusion, stage of treatment, extraction status, and time since appliance activation were not controlled and may have influenced the outcomes. Unequal sample sizes among appliance groups, particularly the smaller combined appliance and fixed retainer subgroups, may have influenced the reliability of between-group comparisons. Although this distribution reflects routine clinical practice, the results related to smaller subgroups should be interpreted with caution. Therefore, the observed differences between appliance types should be considered suggestive rather than definitive. Future multicenter, longitudinal studies with more balanced group sizes are recommended to confirm these findings.

## Conclusion

Fixed orthodontic treatment had minimal impact on adolescents’ day-to-day lives. Most participants reported little or no effect on social and emotional well-being, oral function, or pain. Gender differences were not significant, and longer treatment duration was linked to increased pain.

Although fixed retainers appeared associated with less discomfort while labial appliances with mini-screws and palatal/lingual appliances showed greater impact, these findings should be interpreted cautiously because of the small sample size in some appliance groups.

## Supplementary Information

Supplementary Material 1.

Supplementary Material 2.

Supplementary Material 3.

## Data Availability

The datasets supporting the conclusions of this article are available from the corresponding author upon reasonable request. No public repository currently hosts the clinical data used in this study due to privacy considerations.
