# Supplementary material for: Impact of fixed orthodontic appliances on day-to-day life experiences among adolescents: a cross-sectional observational study
Source: BMC Oral Health. 2026 Jul 6;26:1193. doi: 10.1186/s12903-026-09072-1 (PMC13335211; doi:10.1186/s12903-026-09072-1)
Supplement: Supplementary file 1 — Supplementary Material 1. [file 12903_2026_9072_MOESM1_ESM.pdf]

Orthodontic Treatment Impact Questionnaire

The first section is about how your appliance affects your day-to-day life.

Please circle one response for each of the following questions.

**Think about how your appliance has affected you since your last visit to the orthodontist**			
**Because of my brace or retainer I have been bothered by:**			
	**Doesn't bother me**	**Bothers me a bit**	**Bothers me a lot**
1. Food getting stuck in my brace or retainer	0	1	2
2. Worrying about my brace breaking	0	1	2
3. Difficulty chewing or swallowing	0	1	2
4. Difficulty eating certain foods	0	1	2
**Because of my brace or retainer I have been bothered by:**			
	**Doesn't bother me**	**Bothers me a bit**	**Bothers me a lot**
5. Difficulty pronouncing words	0	1	2
6. Difficulty sleeping	0	1	2
7. Difficulty cleaning my brace or retainer	0	1	2
8. The appearance of my brace or retainer	0	1	2
9. Having my photograph taken	0	1	2
10. Being teased	0	1	2

The second section is about how your appliance feels in your mouth.

Please circle one response for each of the following questions.

**Think about how your brace or retainer has felt in your mouth since your last visit to the orthodontist**
**I have been bothered by my brace or retainer:**

	Doesn’t bother me	Bothers me a bit	Bothers me a lot
11. Feeling tight	0	1	2
12. Rubbing on my gums	0	1	2
13. Making my jaw ache	0	1	2
14. Catching the inside of my mouth	0	1	2

The third section is about how you feel about your appliance.

Please circle one response for each of the following questions.

**Think about how you feel about wearing a brace or retainer since your last visit to the orthodontist**			
**Because of my brace or retainer:**			
	**Don’t feel at all**	**Feel a bit**	**Feel a lot**
15. I feel annoyed	0	1	2
16. I feel shy	0	1	2
17. I feel normal	0	1	2
18. I feel weird	0	1	2
19. I feel ugly	0	1	2
20. I feel attractive	0	1	2
21. I feel negative about my smile	0	1	2

**Overall, thinking about your brace or retainer:**			
	**Doesn’t affect me**	**Affects me a bit**	**Affects me a lot**
22. How does your brace or retainer affect your life overall?	0		
