# Supplementary material for: Impact of fixed orthodontic appliances on day-to-day life experiences among adolescents: a cross-sectional observational study
Source: BMC Oral Health. 2026 Jul 6;26:1193. doi: 10.1186/s12903-026-09072-1 (PMC13335211; doi:10.1186/s12903-026-09072-1)
Supplement: Supplementary file 2 — Supplementary Material 2. [file 12903_2026_9072_MOESM2_ESM.pdf]

استبيان تأثير العلاج التقويمي

(يتم ملؤه من قبل الطبيب)

أرقام مشاركي الدراسة:________________________

تاريخ اليوم:____

نوع ____

الجهاز التقويمي:

تاريخ ____

تركيب الجهاز التقويمي لأول مرة:

شكرًا لك على المشاركة في استبياننا

تذكر أنه لا توجد إجابات صحيحة أو خاطئة؛ نريد فقط معرفة رأيك وما يهمك

يرجى تسليم هذا لموظف الاستقبال أو إرجاعه في الظرف المقدم عند الانتهاء

الجنس **أنثي** ☐ **ذكر** ☐

العمر **سنوات**________ **شهور**__________

هذه الأسئلة تتعلق بكيفية تأثير التقويم عليك وعلى حياتك بشكل عام. بالنسبة لكل سؤال، اقرأ جميع الخيارات وقرر أيها يصف بدقة مشاعرك، ثم احيط بالإجابة المناسبة. يُرجى إعطاء إجابة واحدة فقط لكل سؤال.

الجزء الاول عن كيفية تأثير جهاز التقويم علي حياتك اليومية
من فضلك ضع دائرة حول اجابة واحدة لكل من الاسئلة التالية

**فكر في كيفية تأثير جهازك عليك منذ زيارتك الاخيرة لأخصائي تقويم الاسنان**			
**بسبب جهاز التقويم أو المثبت لقد انزعجت من:**			
	**لا يزعجني**	**يزعجني قليلا**	**يزعجني كثيرا**
١-الطعام يعلق بجهاز التقويم او المثبت	٠	١	٢
٢-القلق من ان ينكسر جهاز التقويم الخاص بي	٠	١	٢
٣-صعوبة المضغ أو البلع	٠	١	٢
٤-صعوبة أكل بعض الطعام	٠	١	٢
**بسبب جهاز التقويم أو المثبت لقد انزعجت من:**			
	**لا يزعجني**	**يزعجني قليلا**	**يزعجني كثيرا**
٥-صعوبة نطق الكلمات	٠	١	٢
٦-صعوبة النوم	٠	١	٢
٧-صعوبة تنظيف جهازالتقويم الخاص بي أو المثبت	٠	١	٢
٨-مظهر جهازالتقويم الخاص بي أو المثبت	٠	١	٢
٩-أخذ صورة فوتوغرافية لي	٠	١	٢
١٠-التعرض للمضايقة	٠	١	٢

الجزء التاني عن كيفية شعورك بالجهاز داخل فمك
من فضلك ضع دائرة حول اجابة واحدة لكل من الاسئلة التالية

**فكر حول شعورك بجهاز التقويم داخل فمك منذ اخر زيارة لأخصائي تقويم الاسنان**			
**لقد انزعجت من جهاز التقويم الخاص بي أو المثبت:**			
	**لا يزعجني**	**يزعجني قليلا**	**يزعجني كثيرا**
١١-الشعور باحكام الجهاز	٠	١	٢
١٢-حكة في اللثة	٠	١	٢
١٣-ألم في الفك	٠	١	٢
١٤-يعلق جهاز التقويم بالأنسجة داخل فمي	٠	١	٢

الجزء الثالث يدور حول شعورك عن جهازك

من فضلك ضع دائرة حول اجابة واحدة لكل من الاسئلة التالية

**فكر فيما تشعر به تجاه ارتداء جهاز تقويم الأسنان أو المثبت منذ زيارتك الأخيرة لأخصائي تقويم الأسنان**			
**بسبب جهاز التقويم أو المثبت:**			
	**لا أشعر علي الاطلاق**	**أشعر قليلا**	**أشعر كثيرا**
١٥-أشعر بالضيق	٠	١	٢
١٦-أشعر بالخجل	٠	١	٢
١٧-أشعر أنني طبيعي	٠	١	٢
١٨-أشعر بالغرابة	٠	١	٢
١٩-أشعر انني قبيح	٠	١	٢
٢٠-اشعر انني جذاب	٠	١	٢
٢١-اشعر بالسلبية تجاه ابتسامتي	٠	١	٢

**في المجمل بالتفكير في جهاز التقويم او المثبت الخاص بك**			
	**لا يؤثر علي**	**يؤثر علي قليلا**	**يؤثر علي كثيرا**
٢٢-كيف يؤثر جهاز التقويم أو المثبت علي حياتك في المجمل	٠	١	٢
