# Supplementary material for: Impact of fixed orthodontic appliances on day-to-day life experiences among adolescents: a cross-sectional observational study
Source: BMC Oral Health. 2026 Jul 6;26:1193. doi: 10.1186/s12903-026-09072-1 (PMC13335211; doi:10.1186/s12903-026-09072-1)
Supplement: Supplementary file 3 — Supplementary Material 3. [file 12903_2026_9072_MOESM3_ESM.pdf]

Dear Editorial Office,

We would like to kindly request a change in the authorship order of our manuscript entitled “[Orthodontic Treatment Impact on Day-to-Day Life Experience of Adolescents with fixed orthodontic appliances: An observational Cross Sectional Study]” (Manuscript ID: 32d8d5bf-c5a8-4018-9be7-9e46709f6166).

Previous order of authors:

Fouad Aly El Sharaby, Nada O. El Zawahry, Shimaa Mohamed G. Ahmed

Revised order of authors:

Shimaa Mohamed G. Ahmed, Nada O. El Zawahry, Fouad Aly El Sharaby

We sincerely apologize for any inconvenience this may cause and appreciate your time and consideration.

Kind regards,

Shimaa Mohamed Gaber Ahmed

On behalf of all co-authors
